# A Symptomatic Killian-Jamieson Diverticulum Detected by Ultrasonography

**DOI:** 10.5334/jbr-btr.877

**Published:** 2015-12-30

**Authors:** M. Navez, P. Bosschaert, J. C. Degols

**Affiliations:** 1 Department of Radiology, Clinique St-Pierre, Ottignies-LLN, Belgium; 2 Department of Otorhinolaryngology, Clinique St-Pierre, Ottignies-LLN, Belgium

**Keywords:** ultrasonography, hypopharyngeal, lateral cervical, Killian-Jamieson divericulum, Zenker’s diverticulum

## Abstract

Killian-Jamieson diverticulum (KJD) is a rare entity resulting from the mucosal herniation through a lateral area of weakness known as the Killian-Jamieson space below the cricopharyngeal muscle. Ultrasonographic diagnosis is exceptional. Moreover, symptoms are found in only eleven percent of patients. KJD in its symptomatic form must be correctly identified because its therapeutic management is in some points different of Zenker’s diverticulum.

A 73-year-old woman was suffering of mild dysphagia with occasional regurgitations and depressible swelling during swallowing on the left side of her neck which appeared two years earlier. Ultrasonographic examination showed a hypoechoic mass surrounded by a digestive wall measuring 45mm located just behind the left thyroid lobe (Fig. A). Moving bright hyperechoic foci caused by air bubbles were clearly observed. Barium swallow pharyngoesophagegraphy highlighted a large protruding sac arising from the left anterolateral pharyngoesophageal junction retaining contrast medium (Fig. B). No concomitant functional abnormalities were found. It was diagnosed as a Killian-Jamieson diverticulum (KJD) based on its specific lateral projection (Fig. C). Computed tomography on procubitus position with oral contrast ingestion was performed demonstrating the diverticulum environment and the absence of complications such as inflammation or fistula.

**Figure A:**
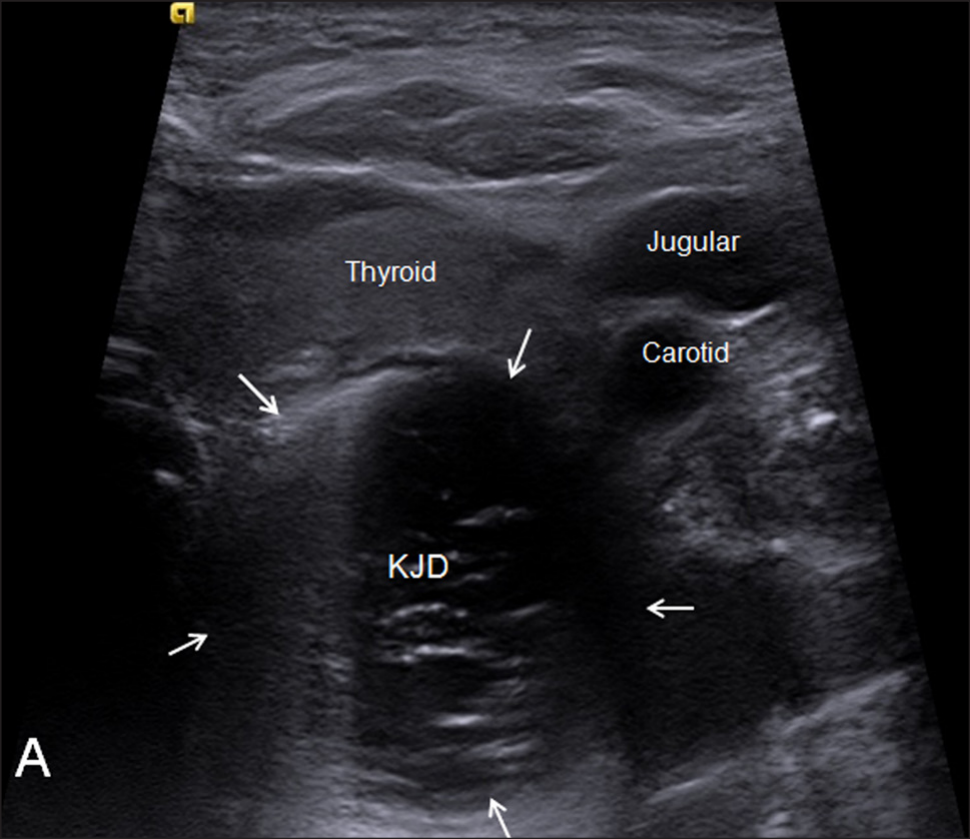
Ultrasonographic axial section showing KJD behind the thyroid left lobe.

**Figure B:**
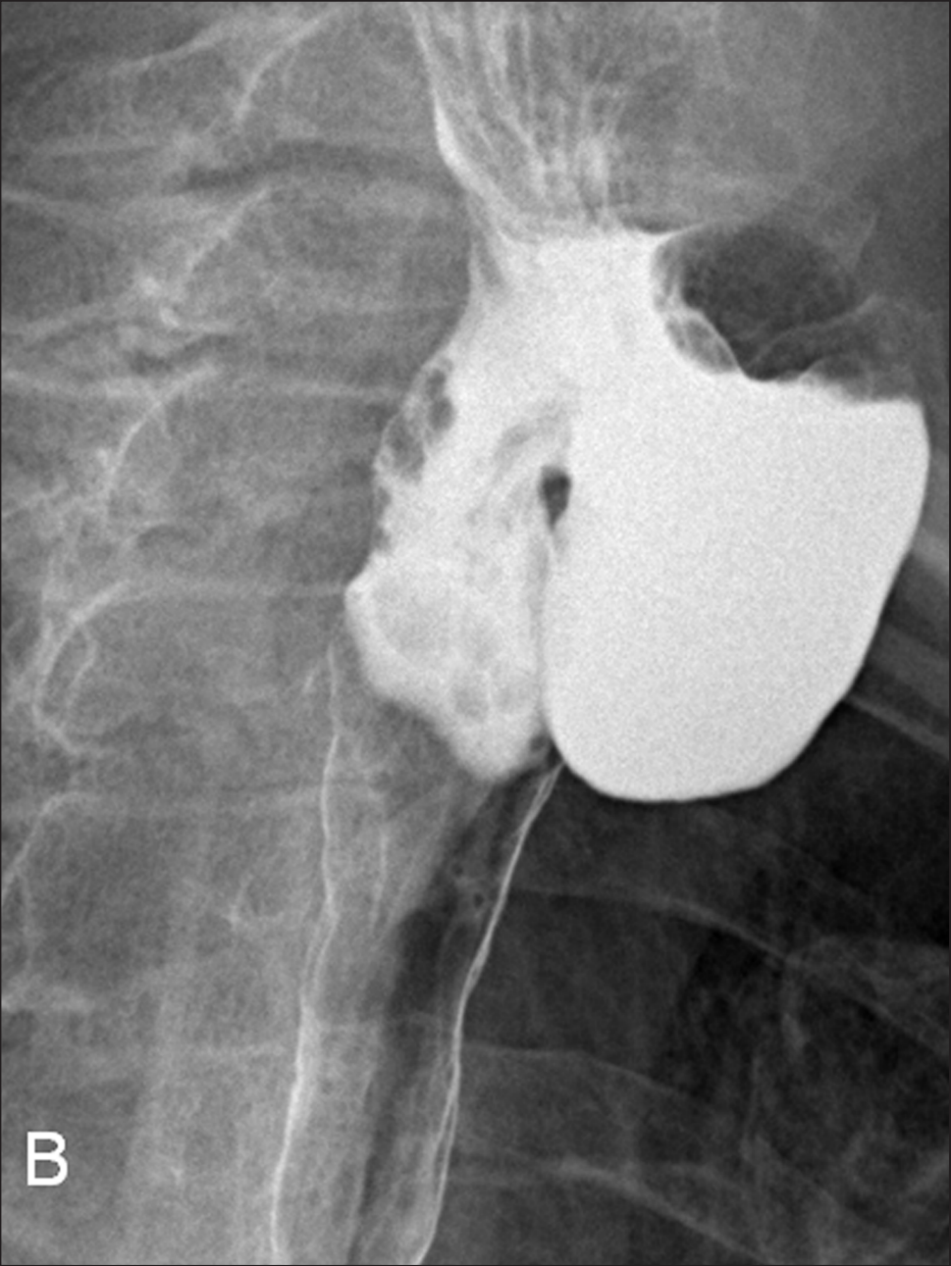
Radiological right anterolateral view after barium administration.

**Figure C:**
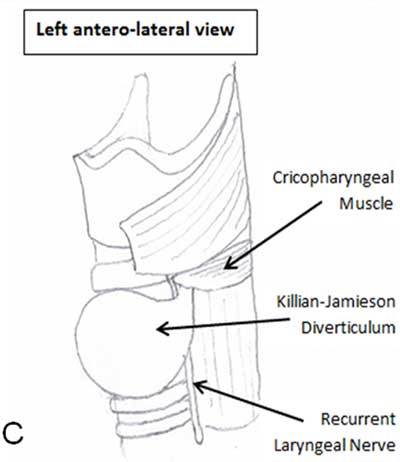
Anatomical drawing.

## Comment

KJD is rare entity related to the more common Zenker’s diverticulum (ZD) with its classic posterior development. It results from the mucosal herniation through a lateral area of weakness known as the Killian-Jamieson space below the cricopharyngeal muscle [1]. This space was first described by G. Killian in 1907 and confirmed by EB. Jamieson in 1934. Seventy-five percent are unilateral on the left side and twenty-five percent are bilateral. Elderly patients are more often affected by this condition. This diverticulum can mimic some thyroid nodules because it is located in the vicinity of the thyroid gland. Symptoms are found in eleven percent of patients. There is no case described of aspiration pneumonia provoked by this abnormality (Rubesin 2001). However, this complication is reported in twelve percent of ZDs. The singular anatomical disposition of the two types of diverticula presumably accounts for the explanation of this difference. Symptomatic KJD may be surgically treated. Although cricopharyngeal myotomy in addition to diverticulotomy is a mandatory component of the ZD treatment, cricopharyngeal myotomy is not necessary for KJD treatment because of the unproved dysfunction of this muscle. We must note here the importance with respect to the directly adjacent recurrent laryngeal nerve entry point into the larynx during the surgical procedure. Endoscopic treatment of this kind of diverticulum has not been found in literature to date and seems inadvisable. Diverticulopexy was only proposed in one case. In conclusion, KJD in its symptomatic form must be correctly identified because its therapeutic management is in some points different of ZD.

## Competing Interests

The authors declare that they have no competing interests.
